# Is the neutrophil a ‘prima donna’ in the procoagulant process during sepsis?

**DOI:** 10.1186/cc13983

**Published:** 2014-07-09

**Authors:** Toshiaki Iba, Takahiro Miki, Naoyuki Hashiguchi, Yoko Tabe, Isao Nagaoka

**Affiliations:** 1 Department of Emergency and Disaster Medicine, Juntendo University Graduate School of Medicine, 2-1-1 Hongo Bunkyo-ku, Tokyo 113-8421, Japan; 2 Department of Clinical Laboratory Medicine, Juntendo University Graduate School of Medicine, 2-1-1 Hongo Bunkyo-ku, Tokyo 113-8421, Japan; 3 Department of Host Defense and Biochemical Research, Juntendo University Graduate School of Medicine, 2-1-1 Hongo Bunkyo-ku, Tokyo 113-8421, Japan

## Abstract

Activation of the coagulation system is a fundamental host defense mechanism. Microorganisms that have invaded the body are trapped and disposed of in clots. Monocytes/macrophages are widely accepted as the main players in the procoagulant process; however, recent evidence suggests that neutrophils also play important roles. Tissue factor, which initiates the extrinsic coagulation cascade, is reportedly expressed on the surface of neutrophils, as well as on microparticles derived from neutrophils. Neutrophil extracellular traps (NETs) are another source of tissue factor. The components of NETs, such as DNA, histones, and granule proteins, also provide procoagulant activities. For instance, DNA initiates the intrinsic pathway, histones are a strong generator of thrombin, and granule proteins such as neutrophil elastase, cathepsin G and myeloperoxidase contribute to the suppression of the anticoagulation systems. Although understanding of the mechanisms that are involved in coagulation/fibrinolysis in sepsis has gradually progressed, the impact of neutrophils on thrombogenicity during sepsis remains to be addressed. Since the importance of the connection between coagulation and inflammation is advocated nowadays, further research on neutrophils is required.

## Introduction

**Figure 1:**
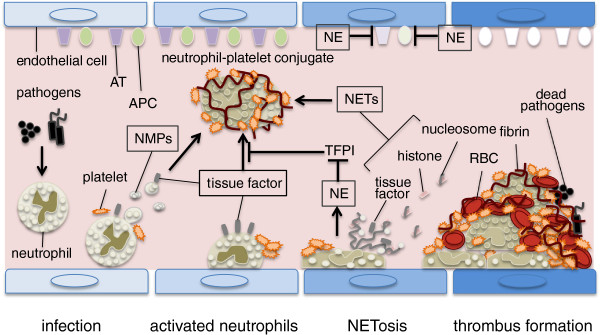
**Neutrophils induce blood coagulation during sepsis.** Neutrophils accumulate and adhere to the vascular endothelium in collaboration with platelets during sepsis. There, they express tissue factor (TF), release TF-bearing neutrophil microparticles (NMPs), and expel neutrophil extracellular traps (NETs) that initiate the coagulation cascade. In addition, neutrophil-derived granule proteins, especially neutrophil elastase (NE), participate in thrombus formation by inhibiting tissue factor pathway inhibitor (TFPI) and anticoagulants such as antithrombin (AT) and activated protein C (APC). Both thrombi formation and endothelial damage lead to substantial microcirculatory damage and organ dysfunction if they occur systemically. RBC, red blood cell.

Activation of the coagulation system is a critical step in the prevention of bacterial dissemination as the coagulation system participates in host defense via fibrin deposition and thrombus formation [1]. Recently, Engelmann and Massberg [2] introduced the term ‘immunothrombosis’ , which designates an innate immune response induced by the formation of thrombi in microvessels and is supported by immune cells and specific thrombosis-related molecules to generate an intravascular scaffold (Figure 1). Immunothrombosis facilitates the recognition, containment, and destruction of pathogens at the infectious site, thereby protecting host integrity without inducing major collateral damage. Once these reactions occur systemically, however, they lead to disseminated intravascular coagulation, and subsequently multiple organ failure and death [3].

Monocytes and macrophages, together with platelets, have long been regarded as the leading actors in activation of the coagulation system; however, recent evidence has suggested that neutrophils also play important roles. For instance, the attenuation of thrombotic manifestations after neutrophil depletion demonstrates the significant contribution of these cells [4]. In the early 1970s Lerner and colleagues [5] suggested a critical role for neutrophils in the thrombotic process, but their theory has only recently been accepted. The cornerstone in this field was a report on neutrophil extracellular traps (NETs), which comprise the extracellular components of neutrophils, including DNA, histones, and neutrophil granule constituents [6]. The observation that NETs express pro-thrombotic and pro-coagulant functions has attracted much attention. In addition to causing microcirculatory disturbances, thrombin generation by neutrophils has been implicated in the induction of further inflammation through the activation of protease activated receptors (PARs) [7,8]. PARs are G-protein-coupled membrane receptors expressed by a variety of cells, including vascular cells. Thrombin and other coagulation factors such as factor VIIa and factor Xa activate platelets and regulate the behavior of other cells through the activation of these receptors. For example, PAR1 is activated when thrombin binds to and cleaves its amino-terminal exodomain to unmask a new receptor amino terminus. This new amino terminus then serves as a tethered peptide ligand, binding intramolecularly to the body of the receptor to effect transmembrane signaling [9]. PARs are also activated by a variety of proteases other than the coagulation factors, such as trypsin and cathepsin G, leading to induction of proinflammatory responses in a variety of cells. Activation of PARs upregulates endothelial expression of tissue factor (TF), mobilization of Weibel Palade bodies and release of von Willebrand factor [10,11]. In this regard, coagulation and inflammation are the two wheels of the host response to infection.

Consequently, knowledge of neutrophil functions is quite important, not only for understanding how the coagulation system is activated but also for elucidating the mechanisms involved in inflammation and host defense. Although anticoagulant therapy could be the treatment of choice for patients with sepsis [12], concurrent effects on neutrophils should be considered. We expect that such approaches may lead to the development of new strategies for controlling severe sepsis.

## Tissue factor expression

TF is a trans-membrane protein that initiates the coagulation cascade and results in thrombin generation [13]. It binds to the coagulation factor VIIa to activate factor X, forming a transient ternary complex. During sepsis, TF is generally accepted to be the main initiator of coagulation. Physiologically, circulating blood is strictly isolated from TF to avoid unexpected clot formation [14]. In contrast, a wide variety of blood cells, as well as endothelial cells, express TF under certain conditions [15,16]. The question arises, however, as to what are the major cellular sources of TF in sepsis? Several cell types that are hemostatically inactive under normal conditions can transform their membrane into a powerful procoagulant surface when exposed to agonists. In particular, endothelial cells and mononuclear phagocytes have been intensively studied and reported to induce synthesis of TF after exposure to stimulating agents during sepsis [17]. Egorina and colleagues [18] reported that lipopolysaccharide induced TF expression in human monocytes *in vitro*. TF expression has also been observed in monocytes from patients with bacteremia [19] or with viral infection [20]. As well as hematopoietic cells, vascular endothelial cells also express TF. One study reported co-localization of TF and von Willebrand factor in the baboon model of sepsis [21].

**Figure 2:**
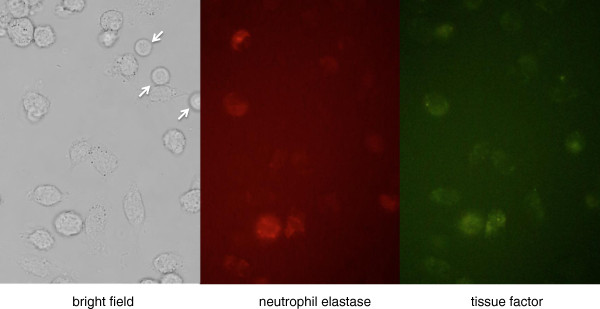
**Tissue factor expression by neutrophils.** Representative micrographs of leukocytes (original magnification 100×). Leukocytes extracted from mouse were stimulated by lipopolysaccharide. Eight hours later, the neutrophils were fixed on the slide and stained with immunofluorescent antibodies to both neutrophil elastase (middle panel) and tissue factor (right panel). The neutrophils were stained by both agents, while lymphocytes (arrows) were negative for the staining.

In contrast, the ability of neutrophils to express TF has been a matter of debate. Although Rapaport and Rao [22] reported in 1995 that TF expressed in neutrophils plays an important role in the activation of coagulation, the significance of neutrophils in thrombus formation had been considered minor. Twenty years have passed since then, and several studies have reported the *in vivo* and *ex vivo* production of TF by neutrophils [23,24]. Maugeri and colleagues [23] and Ritis and colleagues [24] reported that neutrophils produce and express functional TF in response to inflammatory agents, such as C5a, bacterial peptide fMLP and P-selectin. Todoroki and colleagues [25] also reported that TF-positive neutrophils were observed in the circulation in a primate model of sepsis. We also recognized TF expression on the cellular surface of the neutrophils stimulated by lipopolysaccharide (unpublished data; Figure 2). In 2012, Darbousset and colleagues [26] reported that neutrophils are the main source of blood-borne TF using a thrombosis model. Moreover, neutrophils treated with inflammatory mediators reportedly express TF at the mRNA level. On the other hand, Osterud and colleagues [27] reported that *Escherichia coli* phagocytosis in the absence of inflammatory mediators was not sufficient for TF production, despite the observed upregulation of TF mRNA, and this finding is in accordance with the previously reported inability of lipopolysaccharide-treated granulocytes to produce TF [28].

Other studies have also reported that neutrophils do not synthesize TF, but can acquire TF by binding monocyte/platelet-derived microparticles [29]. Regardless of whether TF is produced *de novo* or is acquired from other sources, we think neutrophil-derived TF plays some roles in coagulopathy during sepsis since neutrophils are recognized as a core part of thrombus formation. Indeed, it seems reasonable to consider the activity of TF produced and/or acquired by neutrophils as part of the pathogenesis of thrombotic events during sepsis [30], since neutrophils are the most abundant leukocyte population and enormous numbers of neutrophils take part in the fight against invading microorganisms at the front line of the infection site. Further investigation is required to clarify the major cellular source of septic coagulopathy.

## Neutrophil extracellular trap formation

NETs, which are composed of nuclear and granule constituents of neutrophils, were first described by Brinkmann and colleagues [6]. They primarily entrap and dispose of microbes, thereby contributing to host defense [31]. An alternative effect of NETs is initiation of the coagulation cascade [32,33]. The polyanionic NET surface readily activates contact phase proteins such as factor XII. The contact system (intrinsic cascade) is initiated when blood comes into contact with an anionic surface, and relies on factors intrinsic to flowing blood. In contrast, the extrinsic pathway has received its name because it is initiated by TF, a protein that is not normally in contact with the bloodstream. The participation of the contact pathway is dispensable for physiological hemostasis, and its significance with respect to its role in the activation of coagulation during sepsis is still uncertain. Frick and colleagues [34] reported that the contact system works as a branch of innate immunity and is partly responsible for the activation of sepsis-associated coagulopathy, although many others think that its role is minor. Studies of experimental bacteremia or endotoxemia have shown that activation of coagulation under these circumstances is exclusively mediated by the TF-VIIa route [35]. Blocking contact activation by means of a monoclonal antibody to factor XIIa did not affect *E. coli*-induced disseminated intravascular coagulation in baboons [36]. Thus, further study is needed concerning this issue.

**Figure 3:**
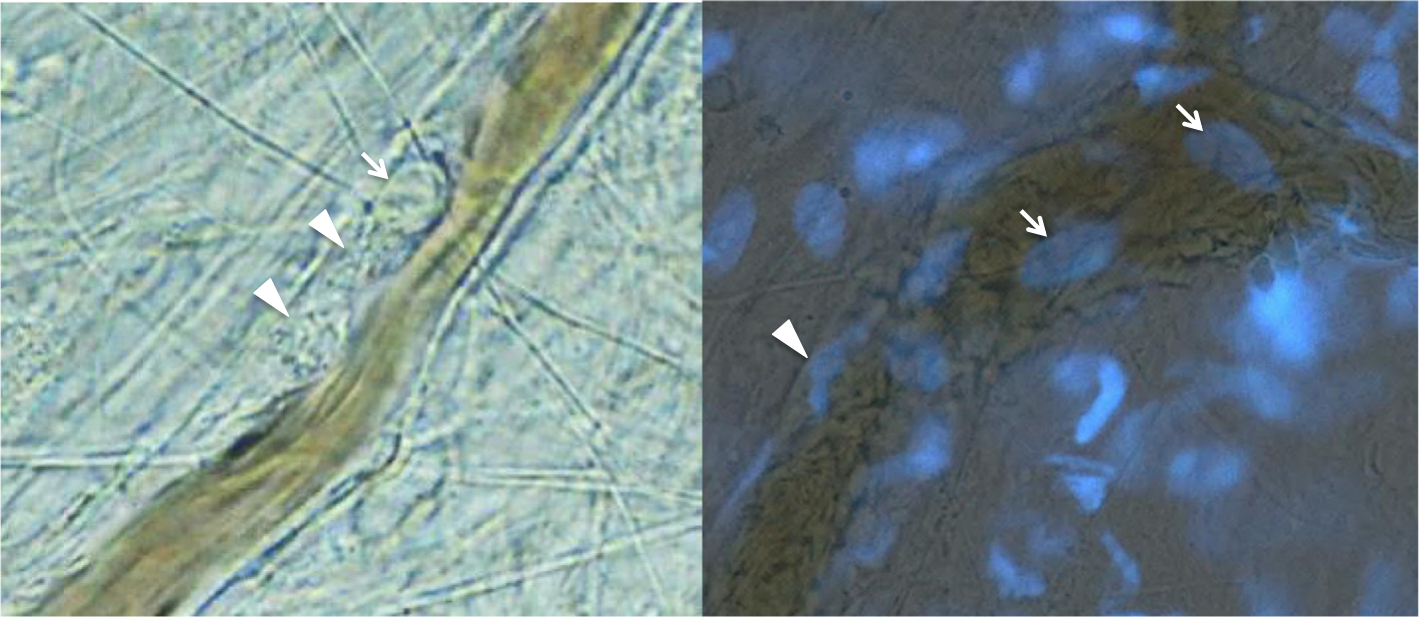
**Neutrophil adhesion to blood vessels.** Left panel: intra-vital microscopic view of the mouse mesenteric vein at 1 hour after lipopolysaccharide infusion. The leukocyte (arrow) adheres to the endothelium and the platelets aggregate in the surrounding area (arrow heads) (objective lens × 20). Right panel: fluorescence live imaging of the mesenteric vein in mouse showing leukocytes in the thrombus (arrows). Fluorescent microscopic examination was performed at 1 hour after infusion of lipopolysaccharide and 4′,6-diamidino-2-phenylindole (DAPI). Nuclei of the damaged endothelial cells were also stained in blue (arrow head) (objective lens × 40).

NETs stimulate platelets via histones H3 and H4 to promote the thrombotic reaction [37]. Furthermore, a part of the mechanism for activation of coagulation is explained by the extracellular delivery of TF by NETs. Kambas and colleagues [38] demonstrated the autophagy-mediated delivery of TF to NETs in sepsis patients. The role of NETs was further examined using experimental models of deep vein thrombosis [39]. These models have provided new information on the initiation and progression of clot formation and the specific impact of NETs; for example, citrullinated histone H3 [40], which is a signature feature of expelling NETs, was observed in thrombi, and the intravenous administration of histones accelerated clot formation [41]. In a mouse model of thrombosis induced by lipopolysaccharide, the earliest event observed in the vessel is the adhesion of leukocytes to endothelium, platelet aggregation and the subsequent endothelial damage (Figure 3). These phenomena indicate the important role of neutrophils in venous thrombus formation [42,43]. The association of TF with NETs can also target thrombin generation and fibrin clot formation to the sites of infection [4]. Kambas and colleagues [38,44] demonstrated that an autophagy-based mechanism is implicated in the extracellular localization of TF in NETs. The linkage between NETs and thrombosis suggests a critical role for neutrophils in the interaction between inflammatory and thrombotic pathways.

**Figure 4:**
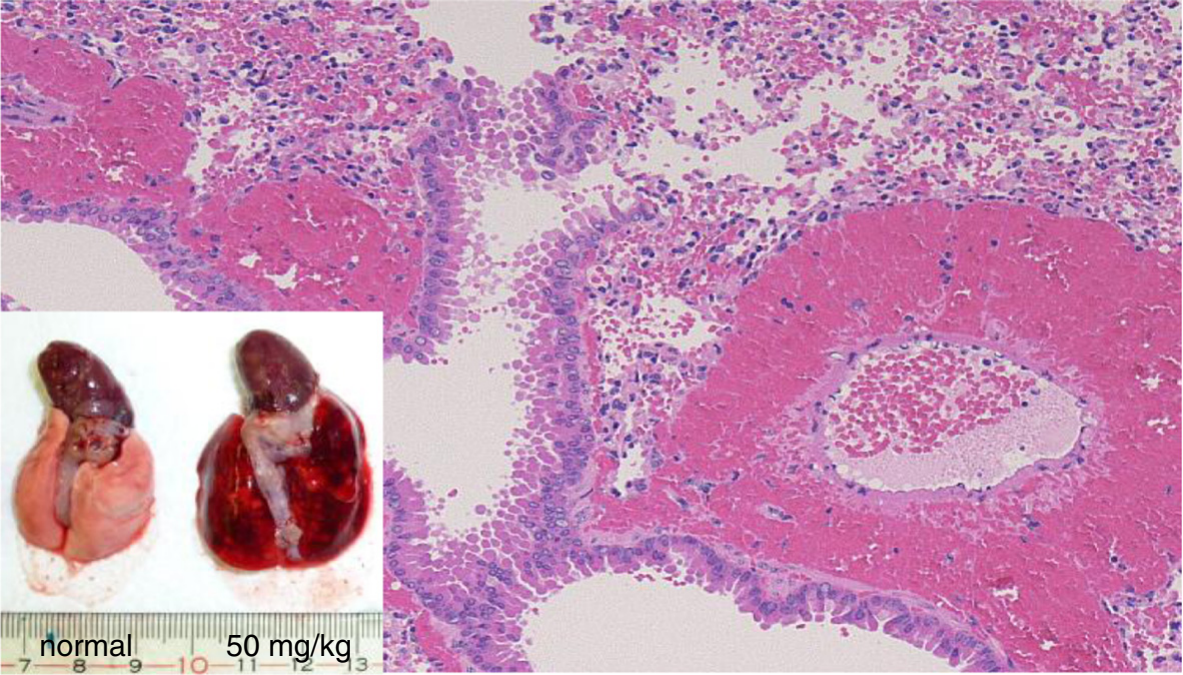
**Histone H3-induced lung injury.** Histological changes in mouse lung 3 hours after 50 mg/kg intravenous histone H3. Macroscopic findings of the lung demonstrate massive bleeding and edema compared to the control (lower left corner). Remarkable bleeding was recognized in the surrounding part of the trachea and the vessels microscopically. (Hematoxylin-eosin staining; ×20 objective lens).

**Figure 5:**
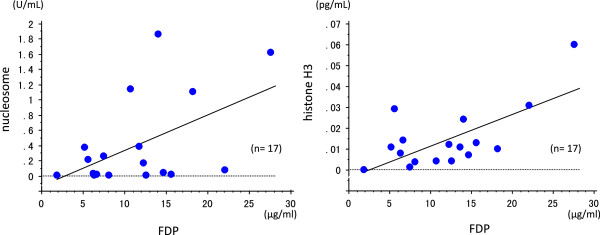
**Relationship between fibrin/fibrinogen degradation products and neutrophil extracellular traps.** Circulating levels of nucleosome and histone H3 were measured in 10 patients (17 points) with severe sepsis. Positive correlation was observed both between nucleosome and fibrin/fibrinogen degradation products (FDP; R^2^ = 0.258), and between histone H3 and FDP (R^2^ = 0.459), suggesting that neutrophil extracellular traps play some role in the activation of coagulation.

As well as TF expression on NETs, histones are also involved in initiation of the coagulation cascade. According to a report by Xu and colleagues [41], histones H3 and H4 have a highly damaging effect on endothelial cells; an intense accumulation of neutrophils is seen in the lungs when histones are injected intravenously into mice, and peri-bronchoalveolar bleeding and thrombus formation is observed, findings that were minimized when an antibody against H4 was administered. We also confirmed the remarkable lung edema, bleeding and thrombus formation in a mouse model after the intravenous administration of histone H3 [45] (Figure 4). Histones are DNA-binding proteins with a positive charge and their toxicity can be diminished by binding of heparins, which are highly sulfated and negatively charged. However, because high-dose heparin increases the risk of bleeding, Wildhagen and colleagues [46] have developed nonanticoagulant heparin, which was reported to improve survival in sepsis.Other circulating NET components, such as nucleosomes and DNA, also activate the coagulation system, and plasma samples obtained from severe sepsis patients demonstrated the positive correlation between the nucleosome and fibrin/fibrinogen degradation products (FDPs), and histone H3 and FDPs (unpublished data; Figure 5).

Histone/DNA complexes are potent activators of human platelets via Toll-like receptor-2 and -4. Histones also interact with the A1 domain of von Willebrand factor, leading to further acceleration of platelet activation [47]. Furthermore, histones induce endothelial cell cytotoxicity and maximize the platelet-endothelial interaction [48]. Two serine protease components of NETs, neutrophil elastase and cathepsin G, also contribute to coagulation (discussed below).

As mentioned above, NET release is thought to play important roles in the pathophysiology of sepsis and activation of coagulation, although it remains controversial whether NETs contribute significantly to sepsis-associated coagulopathy and pathogen killing. To prove the significance of NET release during sepsis, more sophisticated imaging techniques will be needed with appropriate fluorescent dyes to delineate NETs.

As discussed, it is assumed that NETs play at least some role in the development of sepsis-associated coagulopathy; however, the contribution of neutrophils to the activation of coagulation is not limited to infectious diseases. NETs also contribute to coagulopathy in non-infectious diseases such as autoimmune disease [49] and malignancies [50]. Kambas and colleagues [49] demonstrated that the expression of TF in NETs and neutrophil-derived microparticles plays important roles in the induction of thrombosis in active antibody-associated vasculitis.

## Microparticles

Microparticles are released from a variety of cells by budding of the plasma membrane, a process known as ‘ectocytosis’ [51]. These microparticles express the specific antigens of their parental cells [52], and their procoagulant activity depends on negatively charged phospholipids, such as phosphatidylserine, TF, and inclusion proteins [44,53]. Circulating levels of microparticles increase under critical conditions, such as severe sepsis and disseminated intravascular coagulation [54,55]. These reports indicate the substantial increase in interest in the role of microparticles as effectors in the tight tuning of adaptive responses such as inflammation, immunity, and hemostasis [56]. Among microparticles, TF-rich microparticles have been investigated most thoroughly [27,57]. Monocytes/macrophages have been suggested to be the main source of TF-rich microparticles [58], although recent studies have demonstrated that neutrophil-derived microparticles also express TF [44]. In addition, neutrophil-derived microparticles expose phosphatidylserine on their membrane [59] and contain bioactive enzymes such as neutrophil elastase and myeloperoxidase, which all contribute to activation of coagulation [60]. The possibility of neutrophil-derived microparticle-driven coagulation is further supported by a report by Nieuwland and colleagues [61]. They reported elevated levels of neutrophil-derived microparticles in septic patients with disseminated intravascular coagulation and suggested that the coagulation is enhanced by phospholipid and surface TF on neutrophil-derived microparticles. Although the results are not always consistent [60,62], neutrophil-derived microparticles are, at least to some degree, involved in the pathogenesis of coagulation disorder during sepsis.

## Serine proteases

In addition to the aforementioned mechanism of neutrophil-transmitted activation of coagulation, protease-related systems also accelerate coagulation. Neutrophil elastase and cathepsin G are the two major proteases related to coagulation, with neutrophil elastase considered to be dominant. In mice lacking neutrophil elastase but expressing cathepsin G, a significant reduction in fibrin formation has been reported [63]. Neutrophil elastase associated with NETs cleaves tissue factor pathway inhibitor (TFPI) [63,64], a primary inhibitor of TF that is synthesized mainly by endothelial cells and platelets and is presented in the blood as well as on cell surfaces. The increased procoagulant activity induced by the degradation of TFPI is crucial for preventing the spread of invading microorganisms. NET components cooperate efficiently to degrade TFPI, and polyanionic nucleosomes, expelled along with NET ejection, serve as the platform for the binding of neutrophil elastase [63]. In addition, neutrophil proteases have been implicated in the control of platelet responses to vascular injury by enhancing matrix thrombogenicity [65] and also by the cleavage of von Willebrand factor [66]. It is an intriguing scenario that two major bacteriocidal components - the nucleosome and neutrophil elastase - cooperate in clot formation to amplify the entrapment of microbes and facilitate pathogen killing.

Another important function of neutrophil elastase is the inactivation of anticoagulants. Plasma levels of physiological anticoagulants such as antithrombin and activated protein C are known to decrease markedly because of their increased consumption, suppressed synthesis, and degradation by neutrophil proteases [67]. Since the supplementation of these anticoagulants is practically possible and the development of other anticoagulants is currently underway [68], determining the indication, timing and dose for their use is quite important.

## Conclusion

Immunothrombosis is a major element of the intravascular innate immune system. Neutrophils expressing TF are recruited into the circulatory system and the release of TF-bearing neutrophil-derived microparticles, NETs and their components intensifies activation of the coagulation cascade, leading to thrombus formation and the activation of other cell populations, such as platelets and endothelial cells. These cellular and molecular responses all participate in clot formation. In addition, neutrophil-derived proteases, such as neutrophil elastase and cathepsin G, further accelerate these sequential reactions. However, the magnitude of the contribution of neutrophils to the activation of coagulation still remains to be elucidated. The debate on the ability of neutrophils to produce functional TF is ongoing, and the intracellular localization of TF in neutrophils raises a concern regarding its ability to activate the coagulation system. These issues should be addressed in the future. Is the neutrophil a ‘prima donna’ or a ‘corps de ballet’ in the procoagulant process during sepsis? Further evidence is needed to answer this question.

## Abbreviations

FDP: fibrin/fibrinogen degradation product; NET: neutrophil extracellular trap; PAR: protease activated receptor; TF: tissue factor; TFPI: tissue factor pathway inhibitor.

## Competing interests

The authors declare that they have no competing interests.
